# PAHs in baby food: assessment of three different processing techniques for the preparation of reference materials

**DOI:** 10.1007/s00216-015-8490-z

**Published:** 2015-02-03

**Authors:** José Fernando Huertas-Pérez, Luisa R. Bordajandi, Berit Sejerøe-Olsen, Håkan Emteborg, Andrea Baù, Heinz Schimmel, Marta Dabrio

**Affiliations:** 1 European Commission, Joint Research Centre, Institute for Reference Materials and Measurements (IRMM), Retieseweg 111, 2440 Geel, Belgium; 2 Present Address: Department of Analytical Chemistry, Faculty of Sciences, University of Granada, Campus Fuentenueva s/n, 18071 Granada, Spain; 3 Present Address: Unit on Biological Hazards and contaminants, European Food Safety Authority (EFSA), Via Carlo Magno 1/A, 43126 Parma, Italy; 4 Present Address: Unit on Assessment and Methodological Support, European Food Safety Authority (EFSA), Via Carlo Magno 1/A, 43126 Parma, Italy

**Keywords:** PAHs, Baby food, Matrix reference material, Processing

## Abstract

A feasibility study for producing a matrix reference material for selected polycyclic aromatic hydrocarbons (PAHs) in baby food is reported. A commercially available baby food, containing carrots, potatoes, tomato, white beans and meat, was spiked with the so-called 15 + 1 PAHs included in the PAHs priority list for food of the EU, at a mass fraction level of 1 μg/kg. The contaminated baby food was further processed by autoclaving, freezing or freeze drying. The homogeneity of the three materials (bottle-to-bottle variation) and their short-term (4 weeks) and long-term (18 months) stability at different temperatures were assessed. To this end, an analytical method based on a solid–liquid extraction followed by cleaning up with gel permeation chromatography (GPC) and solid phase extraction (SPE) and GC-IDMS determination, was validated in-house. It could be demonstrated that the procedure fulfilled the demands for application to the homogeneity and isochronous stability studies for the candidate reference materials targeted here. All three materials proved to be sufficiently homogeneous for the intended use. Measurements on the autoclaved material provided the most promising results in terms of envisaged shelf life, although freeze drying was also found to be a suitable processing technique for most of the investigated PAHs. These results are an important step towards the development of a matrix reference material for PAHs in a processed food matrix in a presentation very similar to routine samples.

**Electronic supplementary material:**

The online version of this article (doi:10.1007/s00216-015-8490-z) contains supplementary material, which is available to authorized users.

## Introduction

Polycyclic aromatic hydrocarbons (PAHs) are a large class of organic compounds that are composed of two or more fused aromatic rings and are ubiquitous environmental pollutants which can be toxic and carcinogenic depending on the actual compound [1, 2]. PAHs are primarily formed by incomplete combustion or pyrolysis of organic matter (forest fires and volcanoes) and from anthropogenic sources such as motor vehicle exhaust, industrial processes, domestic heating, waste incineration and tobacco smoke [3–5]. Thus, the natural and anthropogenic sources of PAHs in the environment are numerous. Major routes of exposure for non-smokers are food ingestion and air inhalation. Food can be contaminated by PAHs that are present in air, soil or water, by industrial food processing methods (e.g. heating, drying and smoking processes) and during domestic food preparation (e.g. grilling and roasting processes) [6]. As PAHs generally do not occur individually but in mixtures, a set of 16 PAHs were considered by the US Environmental Protection Agency (EPA) as priority pollutants. The 16 EPA PAHs have been commonly used to characterise the PAH load in different samples [7, 8]. However, this list does not include some of the more toxic PAHs as listed by the Scientific Committee on Food (SCF), e.g. 5-methyl chrysene (5-MC), cyclopenta[*cd*]pyrene (CPP), most of the dibenzopyrenes and benzo[*j*]fluoranthene (BjF) [9]. In 2005, the European Commission (EC) listed 15 + 1 PAHs to be monitored by the EU Member States [10], including 15 PAHs identified as genotoxic and carcinogenic by the SCF, together with benzo[*c*]fluorene, which was highlighted by the Joint FAO/WHO Expert Committee on Food Additives (JECFA) [11]. In 2008, the European Food Safety Authority (EFSA) concluded that the sum of either four or eight specific PAHs would be the most suitable indicator for monitoring PAHs in food (noting that monitoring the sum of eight PAHs would not provide a significant added value compared with the sum of four PAHs) [9]. In line with the EFSA opinion, current EU legislation sets maximum concentration levels for the sum of four PAHs (benzo[a]pyrene, benzo[a]anthracene, benzo[b]fluoranthene and chrysene) to minimise the health risk from dietary PAHs exposure [12].

Liquid chromatography coupled to fluorescence detection (LC-FLD) and gas chromatography coupled to mass spectrometry (GC-MS) are the most commonly used techniques for instrumental determination of PAHs in complex food and environmental samples [5, 6]. In the past, LC with ultraviolet (UV) or photo-diode array (PDA) detector [13, 14] and GC with flame ionisation detector (FID) were widely applied [9], although they lack of sufficient sensitivity and selectivity. It is possible by using LC-FLD to separate and determine some PAH isomers which are difficult to separate by GC, such as benzo-fluoranthenes or dibenzo-anthracenes [6, 8]. A better performance for the heaviest compounds is achieved as well, since it avoids discrimination observed for these compounds during GC injection at high temperatures [15, 16]. Nevertheless, GC has become the preferred technique for PAH analysis as its coupling with MS detection provides superior resolution capabilities, improved sensitivity and selectivity as well as the possibility of using isotopically labelled internal standards [16, 17]. Sample preparation for PAH determination in complex solid food matrices usually involves solid–liquid extraction (SLE) and a subsequent clean-up step to avoid interfering compounds and to minimise the maintenance of the chromatographic system, especially when using GC [6]. Different techniques such as Soxhlet extraction, pressurised liquid extraction (PLE) and ultrasound-assisted extraction have been successfully applied for a wide range of matrices, while gel permeation chromatography (GPC), solid phase extraction (SPE) and combinations thereof are the most popular strategies to clean the extracts [6].

Certified reference materials (CRMs) are crucial quality assurance tools for laboratories to validate and operate measurement procedures, in order to deliver reliable and accurate results. Guidelines for the production and certification of RMs are provided in ISO Guide 34 [18] and ISO Guide 35 [19] and are adopted by corresponding CRMs producers, which are in some cases also accredited for the activity. Several challenges must be considered for the preparation of CRMs. For example, the between-bottle heterogeneity must be quantified and sufficiently low as well as the short- and long-term stability of the target parameters must be investigated. Moreover, suitable and representative analyte/matrix combinations that are similar to routine samples with respect to mass fraction levels and material properties have to be selected. Incurred materials are generally preferred as starting materials because of their proximity to real samples, however they are often difficult to obtain. Spiked materials could be a suitable alternative as long as sufficient representativeness, equivalence, homogeneity and stability are achieved for the candidate CRM. During the material processing, the minimisation of analyte and matrix modifications is desirable in order to preserve the material properties as much as possible. The production of CRMs is a very complex process and requires a thorough planning. Therefore, prior feasibility studies are frequently required to investigate the best way of preparing the material to ensure a sufficiently homogeneous and stable CRM, which will be suitable for its purpose [19].

This work assesses the feasibility of producing a matrix RM for the 15 + 1 EU priority PAHs, at a concentration level of 1 μg/kg, using a commercially available baby food as starting material. Different processing procedures such as freezing and freeze drying, traditionally employed for preparing matrix reference materials, and the more novel autoclaving procedure were considered with three main objectives: (i) to obtain materials with minimal manipulation (i.e. as close as possible to real samples) avoiding PAH degradation; (ii) sufficient material homogeneity; and (iii) sufficient stability of the target parameters upon storage and shipment of the material. In order to evaluate the homogeneity and stability of the three resulting materials, a fit-for-purpose method for the determination of the 15 + 1 EU PAHs has been validated in-house. It was especially relevant to achieve highly repeatable results since they increase the sensitivity of the tests applied to detect potential heterogeneous distribution of PAHs in the baby food materials. The same is true during the assessment of the PAHs stability via isochronous studies, where analytical measurements are performed under repeatability conditions.

## Experimental

### Chemicals and materials

PAHs as neat solids used in this study (Table 1) were either from BCR- or ERM-certified reference materials provided by the Institute for Reference Materials and Measurements (IRMM; Geel, Belgium), except cyclopenta[*cd*]pyrene (CPP), obtained from the Biochemical Institute for Environmental Carcinogens (Grosshansdorf, Germany), benzo[*c*]fluorine (BcF) and dibenzo[a,i]pyrene (DaiP) which were obtained from Dr. Ehrenstorfer (Augsburg, Germany). Solutions used for spiking the material were gravimetrically prepared using acetonitrile as solvent. All other solutions, i.e. individual stock solutions, a mixed stock solution containing all PAHs and subsequent working solutions for the analytical calibration were gravimetrically prepared using toluene as solvent. Isotopically labelled compounds with stated chemical purity benzo[*a*]anthracene-D_12_ (BaA-D_12_; 99.5 %), chrysene-D_12,_ (Chr-D_12_; 99.5 %), benzo[*b*]fluoranthene-D_12_ (BbF-D_12_; 99.0 %), benzo[*k*]fluoranthene-D_12_ (BkF-D_12_; 99.5 %), benzo[*a*]pyrene-D_12_ (BaP-D_12_; 99.0 %), indeno[*1,2,3-cd*]pyrene-D_12_ (IcdP-D_12_; 100 %), dibenzo[*a,h*]anthracene-D_14_ (DahA-D_14_; 99.0 %) benzo[*g,h,i*]perylene-D_12_ (BghiP-D_12_; 98.5) and dibenzo[a,i]pyrene-D_14_ (DaiP-D_14_; 99.0 %) were purchased as neat crystals from Dr. Ehrenstorfer (Augsburg, Germany) and used as internal standards.

**Table 1:** 15 + 1 EU PAHs used in the feasibility study

PAH	Abbreviation	CAS number	BCR®/ERM® code	Purity (g/g)	*U* ^a^ (g/g)
Benzo[*c*]fluorene	BcF	205-12-9	–^b^	0.982^c^	
Benz[*a*]anthracene	BaA	56-55-3	BCR®-271	0.9984	0.0009
Chrysene	Chr	218-01-9	BCR®-269	0.9928	0.0028
Cyclopenta[*cd*]pyrene	CPP	27208-37-3	–^b^	0.996^c^	
5-methylchrysene	5-MC	3697-24-3	BCR®-081R	0.9973	0.0013
Benzo[*b*]fluoranthene	BbF	205-99-2	BCR®-047	0.9974	0.0026
Benzo[*k*]fluoranthene	BkF	207-08-9	BCR®-048R	0.997	+0.003
					−0.004
Benzo[*j*]fluoranthene	BjF	205-82-3	BCR®-049	0.997	+0.003
					−0.006
Benzo[*a*]pyrene	BaP	50-32-8	ERM®-AC051	0.973	0.013
Indeno[1.2.3-*cd*]pyrene	IcdP	193-39-5	ERM®-AC053	0.996	+0.004
					−0.005
Dibenz[*a.h*]anthracene	DahA	53-70-3	BCR®-138	0.990	0.007
Benzo[*ghi*]perylene	BghiP	191-24-2	BCR®-052	0.9923	0.0021
Dibenzo[*a.l*]pyrene	DalP	191-30-0	BCR®-096	0.9972	0.0025
Dibenzo[*a.e*]pyrene	DaeP	192-65-4	BCR®-133	0.996	+0.004
					−0.005
Dibenzo*[a.i]*pyrene	DaiP	189-55-9	–^b^	0.996^c^	
Dibenzo*[a.h]*pyrene	DahP	189-64-0	BCR®-159	0.993	0.007

^a^The uncertainty is the expanded uncertainty estimated in accordance with the Guide to the Expression of Uncertainty in Measurements (GUM) with a coverage factor *k* = 2, corresponding to a level of confidence of about 95 %

^b^Commercial material

^c^Purity of the analytical standard as stated by the provider

Acetonitrile (MeCN), toluene, dichloromethane (DCM) and *n*-hexane used in this study were of SupraSolv grade from Merck KGaA (Darmstadt, Germany). Sodium sulphate (≥99.0 %) was obtained from Sigma, and Accubond™ AminoII SPE cartridges (6 mL, 1 g) were obtained from Agilent Technologies (PaloAlto, CA, USA).

### Preparation of the study materials

**Fig. 1:**
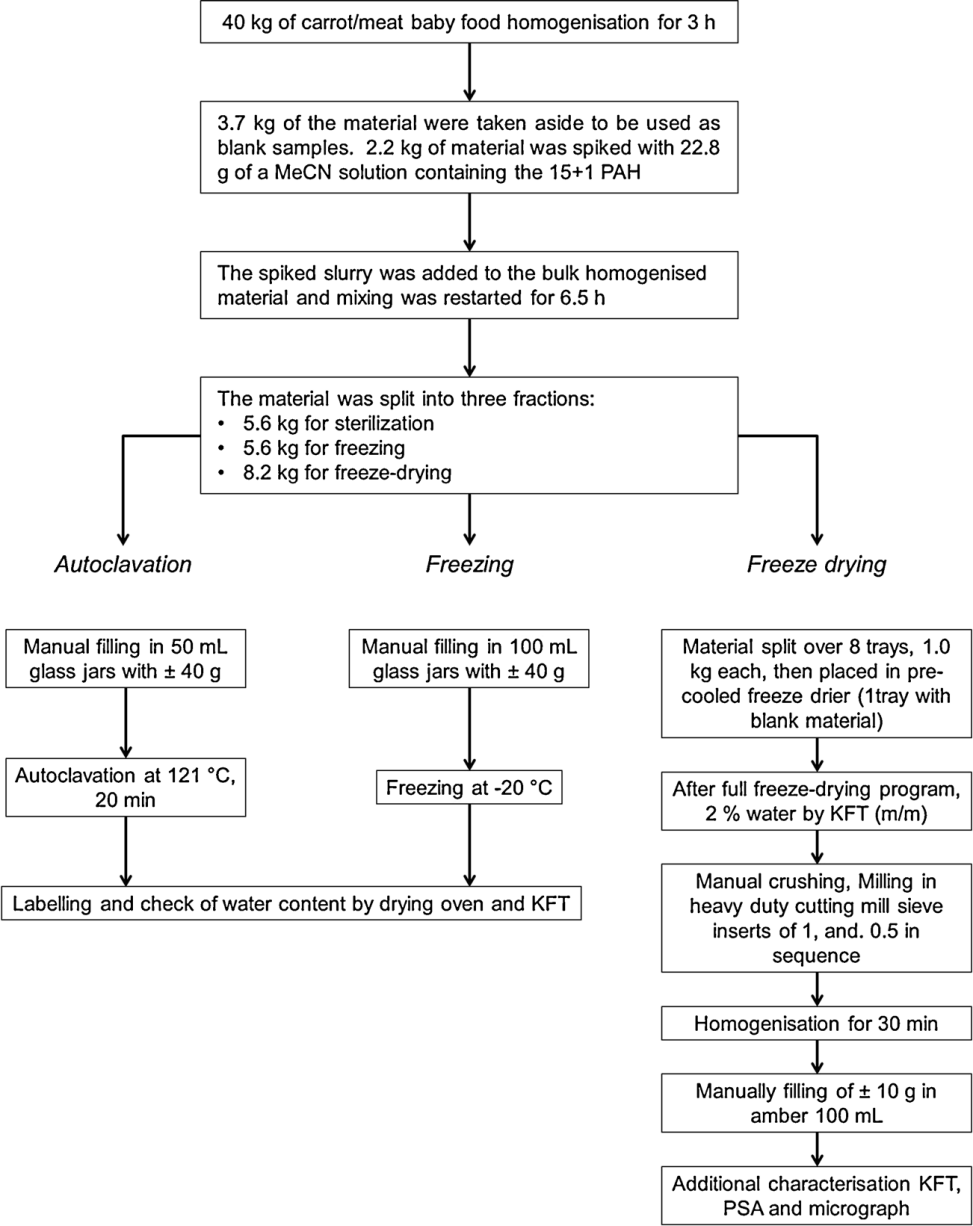
Flow diagram for the preparation of the three materials considered in this study

Figure 1 shows a flow diagram for the preparation of the three materials considered in this study. The content of 202 jars of commercially available baby food (40 kg of material) was placed in a stainless steel mixing vessel of a paste mixer (IKA-Janke Kunkel, Staufen, Germany) and mixed at full speed for 3 h. The baby food had a composition of 40 % carrots, 18 % potatoes, 18 % tomatoes, 13 % white beans, 10 % meat and 1 % maize oil, as stated by the product label, and was supplied as a very fine homogeneous paste. The direction of mixing was changed every 30 min. Next, 3.7 kg of the material were taken aside in a stainless steel container for blank samples. From the remaining material, a portion of 2.2 kg was placed into a glass beaker and thoroughly mixed with 22.8 g of a spiking solution, containing the 15 + 1 priority PAHs in MeCN at a mass fraction of approximately 2.2 μg/g each. The mixture was stirred manually with a glass rod and subsequently added to the rest of the baby food and homogenised for 6.5 h. The target mass fraction was 1 μg/kg for each PAH, as it is the maximum level established by the current EU regulation for BaP in baby food [12]. Once the material was thoroughly mixed it was divided in three parts for further treatment resulting in three different matrix presentations.

#### Frozen material

Forty-gramme portions of the homogenised material were manually filled into 100 mL glass jars (140 spiked plus 10 blank units), closed with screw cap and subsequently introduced in aluminium pouches thermally sealed to avoid light exposure. The material was stored at −20 °C until analysis.

#### Autoclaved material

Forty-gramme portions of the homogenised material were manually filled into 50 mL glass jars (140 spiked plus 10 blank units), which were closed with screw cap and introduced in an autoclave system (Matachana B4023, Webeco, Ober-Ramstadt, Germany ) for sterilisation at 121 °C for a period of 20 min, with a total cycle time of 1 h. Once the cycle was finished and room temperature was reached, the jars were put into aluminium pouches thermally sealed, in order to avoid light, and stored at 4 °C until analysis.

#### Freeze-dried material

Approximately 8 kg of the homogenised material was spread on Teflon-coated trays (1.0 kg/tray) and freeze dried in an Epsilon 2-85D freeze-dryer (Martin Christ, Osterode, Germany) for 70 h. The initial starting temperature was −30 °C, and the sublimation step lasted about 60 h with step-wise increments of shelf temperature by 10 °C at a time. Following a secondary drying step at 20 °C for about 8 h, the material was sufficiently dry. The freeze-dried material was manually crushed with a PFTE pestle and then milled with a heavy duty cutting mill (Retsch, Haan, Germany) with a 1.0 mm sieve insert. In order to perform a second milling with a 0.5-mm sieve insert, a prior cooling of both the material and the mill with liquid N_2_ was necessary. The powder obtained after milling was homogenised in a Dyna-MIX CM200 mixer (WAB, Basel, Switzerland). Ten-gramme portions were filled into 100 mL amber glass vials (140 spiked plus 10 blank units) and closed with an insert and aluminium crimp caps. The material was stored at 4 °C until analysis.

### Analytical methodology for determination of PAHs

#### Preparation of calibration standards

Individual standard stock solutions of both native and isotopically labelled compounds were prepared gravimetrically with a PAH mass fraction of approximately 50 μg/g in toluene and kept in 100 mL capped amber vials at −20 °C until use. Two intermediate stock solutions, one containing the native compounds and the other containing the isotopically labelled compounds, were prepared by mixing appropriate amounts of each individual stock solutions and toluene to reach a mass fraction of approximately 1500 μg/kg of each PAH. Appropriate amounts of each intermediate stock solution were mixed and, once again, diluted gravimetrically with toluene to obtain calibration working solutions covering the range 50–150 μg/kg of each native compound and containing 100 μg/kg of each isotopically labelled compound.

#### Sample treatment procedure

Sample treatment was based on procedures described elsewhere with some modifications [20–23]. It comprised an ultrasound-assisted solid–liquid extraction and a subsequent thorough clean-up based on GPC followed by SPE. As soon as the frozen (FR) and autoclaved (AC) materials reached room temperature, they were homogenised and 10-g aliquots were weighed into 50-mL glass tubes. For the freeze-dried (FD) material, 2 g were weighed into the glass tubes (the mass reduction because of water loss during freeze-drying process is 81.2 %, i.e. a pre-concentration of about five times), and 8 g of MilliQ water was gravimetrically added. In both cases, the contents were thoroughly mixed before and after addition of an appropriate amount of the IS solution to reach a mass fraction of approximately 1 μg/kg in the baby food. The samples were allowed to equilibrate for at least 1 h at room temperature and in the dark before extraction. Then, 10 mL of DCM were added, sonicated on an ultrasonic cleaner (USC600TH, VWR, Leuven, Belgium) for 5 min and then centrifuged (Heraeus Megafuge 1.0RS, Kendro Laboratory Products GmbH, Hanau, Germany) at 4000 rpm for 5 min at 18 °C. After centrifugation, three different layers were obtained. The upper aqueous layer and the middle solid layer were discarded, and the organic layer, located at the bottom of the tube, was transferred to a 50-mL glass tube with the help of a Pasteur pipette. The extract was mixed with two spoons of Na_2_SO_4_, filtered through a 0.45-μm PTFE syringe filter directly into a 10-mL GPC vial and concentrated to approximately 2.5 mL under a gentle stream of nitrogen before injection into a GPC system (AccuPrep MPS™ GPC system, J2 Scientific, Columbia, MO, USA, equipped with a Express™ column (J2 Scientific). DCM was used as organic mobile phase at a flow rate of 3 mL/min. The fraction from 24 to 36 min was collected (36 mL) in a round flask and evaporated down to approximately 1 mL by means of a rotary evaporator (40 °C, 750 mbar). After that, 1 mL of *n*-hexane was added, and a second clean-up step was performed loading the extract into SampliQ Amino (NH_2_) SPE cartridge (6 mL, 1000 mg, Agilent). The SPE cartridge was previously conditioned with 20 mL of *n*-hexane/DCM (1:1, *v*/*v*). Twenty-five millilitres of *n*-hexane/DCM (1:1, *v*/*v*) were passed through the cartridge for analyte elution. The fraction was collected and subsequently pre-concentrated under a gentle nitrogen stream. Finally, 100 μL of toluene were added, and the extract was transferred to an amber glass vial for injection into the chromatographic system.

#### GC-MS analysis

The GC-MS system consisted of a Thermo Trace GC system (Thermo-Finnigan, Milan, Italy) coupled to a Trace DSQ single quadrupole mass spectrometric detector (Thermo-Finnigan). Injections (injection volume, 1 μL) were automatically performed by means of a PAL autosampler (CTC Analytics, Zwingen, Switzerland), into a split/splitless programmed-temperature vaporising injector (PTV) using a 10 μl syringe. The mass spectrometer was operated in the electron ionisation (EI) mode (electron energy, 70 eV). GC-MS settings were the same as reported elsewhere [17]. The GC separation was performed using a fused silica DB-17HT (50 % phenyl methyl polysiloxane, 30 m length, 0.32 mm i.d., 0.15 μm film thickness) from Agilent Technologies. Helium at 99.9997 % (AirLiquide, Belgium) was used as carrier gas at a flow rate of 1.5 mL/min. The PTV injector temperature was held at 110 °C for 0.01 min, ramped to 330 °C at 8 °C/s and held at this temperature for 5 min. Subsequently, the injector was purged during 8 min at a temperature of 350 °C and a flow rate of 100 mL/min. The oven temperature was programmed as follows: 80 °C held for 1.5 min, ramped to 250 °C at 10 °C/min, held for 5 min and finally ramped to 320 °C at 10 °C/min and held for 10 min. The GC-MS ion source temperature was 250 °C and the transfer line temperature was 320 °C. The quadrupole temperature was set to 150 °C. Each target compound was monitored in the selective ion monitoring mode (SIM) according to their retention time, using one quantitative ion (QT) and one qualitative ion (QL). The programmed time-segmented group and related selected ions monitored for each selected PAHs are shown in Table 2.

**Table 2:** GC-MS parameters for the determination of PAHs

PAH	MW	Retention time (min)	(*m/z*)		QL ion (% relative abundance)	Time window (start time (min))
			QT ion	QL ion		
BcF	216	16.62	216	215	82.0	13.00
BaA	228	18.54	228	229	19.7	
BaA-D12	240	18.48	240			
Chr	228	18.75	228	229	20.0	
Chr-D12	240	18.68	240			
CPP	226	18.68	226	224	24.0	
5-MC	242	20.05	242	241	60.0	
BbF	252	22.26	252	250	24.3	21.50
BbF-D12	264	22.14	264			
BkF	252	22.38	252	250	23.7	
BkF-D12	264	22.28	264			
BjF	252	22.59	252	250	28.6	
BaP	252	24.25	252	250	24.7	
BaP-D12	264	24.19				
IcdP	276	27.84	276	277	22.5	26.50
IcdP-D12	288	27.77	288			
DahA	278	27.96	278	276	23.3	
DahA-D14	292	27.87	292			
BghiP	276	28.64	276	277	22.1	
BghiP-D12	288	28.57	288			
DalP	302	31.14	302	300	50.0	30.00
DaeP	302	31.76	302	300	25.6	
DaiP-D14	316	32.07	316			
DaiP	302	32.17	302	300	20.3	
DahP	302	32.44	302	300	20.9	

*QT* quantifying ion, *QL* qualifying ion

#### Method validation

The analytical method was applied for homogeneity and stability studies and was validated in-house. The performance parameters evaluated included: selectivity, linearity and working range, detection (LOD) and quantification limits (LOQ), trueness, method repeatability and stability of the extracts. Each day of the validation, a method blank (no matrix) was prepared following the procedure described in “Sample treatment procedure” and analysed in order to check for any possible reagent or material cross-contamination.

Blank samples of each material were also checked for the presence/absence of any potential interfering peaks. A set of solvent standards at mass fraction levels of 50, 75, 100, 125 and 150 μg/kg of each PAH, and 100 μg/kg of each internal standard, was prepared every day and injected five times along the measurement sequence. The quantification of analytes in the samples was carried out with solvent standards according to Eq. (1). A relative response factor (RRF) was applied as average value of the RRFs, obtained from the three calibration data sets for each concentration level (5 concentration levels × 5 instrumental replicates × 3 days of the validation study) according to Eq. (2).

1 $$ {w}_{{\mathrm{PAH}}_x}=\frac{A_{{\mathrm{PAH}}_x}*{w}_{{\mathrm{d}\hbox{-} \mathrm{P}\mathrm{A}\mathrm{H}}_x}*{m}_{{\mathrm{d}\hbox{-} \mathrm{P}\mathrm{A}\mathrm{H}}_x}}{A_{{\mathrm{d}\hbox{-} \mathrm{P}\mathrm{A}\mathrm{H}}_x}*\mathrm{R}\mathrm{R}\mathrm{F}*{m}_{\mathrm{sample}}} $$

2 $$ \mathrm{R}\mathrm{R}\mathrm{F}=\frac{A_{{\mathrm{PAH}}_x}*{w}_{{\mathrm{d}\hbox{-} \mathrm{P}\mathrm{A}\mathrm{H}}_x}}{A_{{\mathrm{d}\hbox{-} \mathrm{P}\mathrm{A}\mathrm{H}}_x}*{w}_{{\mathrm{PAH}}_x}} $$

Where ${w}_{{\mathrm{PAH}}_x}$ is mass fraction of the PAH_*x*_ (μg/kg); ${A}_{{\mathrm{PAH}}_x}$ is area of the PAH_*x*_; ${A}_{{\mathrm{d}\hbox{-} \mathrm{P}\mathrm{A}\mathrm{H}}_x}$ is area of the deuterated PAH_*x*_; ${w}_{{\mathrm{d}\hbox{-} \mathrm{P}\mathrm{A}\mathrm{H}}_x}$ is mass fraction of deuterated PAH_*x*_ (μg/kg); ${m}_{{\mathrm{d}\hbox{-} \mathrm{P}\mathrm{A}\mathrm{H}}_x}$ is mass of the deuterated mix solution added to the vial (g); and *m*_sample_ is mass of sample taken for analysis (g).

In order to study method linearity and working range for each analyte, calibration curves were constructed by plotting the peak area ratio (native/isotopically labelled) versus the mass fraction ratios (native/isotopically labelled), obtained from standard solutions. Linearity was firstly evaluated by visual inspection of the plotted data (area ratio and mass fraction ratio of analytes and isotopically labelled standards), and then by the residual plots and calculation of the correlation coefficient (*r*). Linearity was also assessed through the RSD of the average RRFs obtained each day. In addition, *p* values for the lack-of-fit test at 95 % confidence interval (*α* = 0.05) were obtained using Statgraphics 5.0. Software [24]. LOD and LOQ were estimated through the calibration data according to the German Standard DIN 32645 [25]. Trueness was assessed by spiking experiments at two different mass fraction levels, i.e. 1 and 1.5 μg/kg for frozen and autoclaved materials and five times higher mass fraction for the freeze-dried material (5 and 7.5 μg/kg) due to the pre-concentration because of water loss during freeze drying. For this purpose, three different blank samples of each material were spiked at each concentration level, processed according to the abovementioned sample preparation procedure and injected twice. Then, the recovery values were calculated by comparing both the measured and the theoretical mass fractions of PAHs. The repeatability of the method was assessed for each material type by analysing six samples per material in duplicate (*n* = 12). Each subsample was injected twice on the GC. The stability of the extracts was investigated by injecting spiked samples (at 1 μg/kg) at the day of preparation and re-analysing them after 2 weeks. During that period, the extracts were kept in closed (capped) vials and stored at +4 °C in darkness.

### Homogeneity studies

Equivalence between all the bottles produced in a batch is an essential requirement for any reference material (RM). Therefore, RM producers are required to quantify the between-bottle heterogeneity of the certified parameters [19]. For each material, eight bottles were chosen using a random stratified sample selection scheme, thus ensuring that the complete batch was covered. Three independent replicate determinations per sample were performed for their PAH content by applying the in-house-validated analytical methodology. The measurements were carried out under repeatability conditions (in one analytical run) and in a random order to allow to separate analytical drift and a possible trend in the filling sequence. The data were evaluated using analysis of variance (ANOVA) via the SoftCRM software [26]. Linear regression analyses were performed for all the PAH results to detect any potential trends in the filling sequence. Grubbs tests at 99 % confidence levels were employed to identify potential outliers among individual replicate results or bottle averages.

The distribution of results was checked using normal probability plots and histograms. Finally, ANOVA was performed to calculate the within-bottle standard deviation (*s*_wb_) and the between-bottle standard deviation (*s*_bb_). As *s*_wb_ and *s*_bb_ are estimates of the true standard deviations subject to random fluctuations, the mean square between groups (MS_between_) could be smaller than the mean squares within groups (MS_within_). In these cases, *s*_bb_ cannot be calculated, and the maximum heterogeneity that could be hidden by method repeatability (*u* ^*^_bb_), is an alternative to estimate the uncertainty contribution due to possible heterogeneity. *u* ^*^_bb_ was estimated as described elsewhere [27].

### Stability studies

Stability testing is necessary to (i) establish suitable conditions for material dispatch to the end user (short-term stability study) and to (ii) establish suitable conditions for the storage of a CRM (long-term stability study). Knowledge of the stability during transport, covering the possible worst-case scenarios, is necessary in order to ensure that the potential instability of the material during dispatch is negligible [19].

Stability studies were conducted following isochronous schemes [28]. The samples were moved to a reference temperature after different periods of time at the temperatures evaluated. The design allows the simultaneous analysis of the samples at various exposure times, i.e. under repeatability conditions, thus greatly improving the sensitivity of the study to detect degradation and increasing the significance of the result because of the reduction of the analytical variation. For both studies (short- and long-term), six independent sub-samples (2 bottles × 3 replicates) were analysed for each time/temperature point. Storage temperatures for the short-term stability study were −20, 4 and 18 °C (FR batch) and 4, 18 and 60 °C (AC and FD batches). For the long-term stability study, temperatures were −20 °C (FR batch) and 4 and 18 °C (AC and FD batches). The reference temperature was −70 °C for the frozen batch and −20 °C for the autoclaved and freeze-dried batches. Storage times were 0, 1, 2 and 4 weeks (short-term stability study) and 0, 6, 12 and 18 months (long-term stability study). Measurements were carried out by applying the in-house-validated analytical procedure. The results were grouped and evaluated per time point and temperature and screened for outliers. Linear regression analysis as a function of time was performed to check for significant trends which may indicate degradation of the analytes. The slopes were tested for significance using a *t* test with *t*_*α*, df_ being the critical *t* value (two-tailed) for a significance level *α* = 0.01 (99 % confidence interval). The uncertainties associated to short-term stability (*u*_sts_) and long-term stability (*u*_lts_) were estimated according to the following equation [29], for periods of 4 weeks (short-term study) and 18 months (long-term study), respectively:

3 $$ {u}_{\mathrm{rel}\ \left(\mathrm{s}\mathrm{t}\mathrm{s}\kern0.5em \mathrm{or}\ \mathrm{l}\mathrm{t}\mathrm{s}\right)}=\frac{{\mathrm{RSD}}_{\mathrm{stab}}}{\sqrt{{\displaystyle \sum \Big({x}_{\mathrm{i}} -}\overline{x}\Big){}^2}}\ast {t}_{\mathrm{sl}} $$

where RSD_stab_ is the relative standard deviation of all results of the stability study, *x*_i_ is the time point for each replicate, $\overline{x}$ is the average of all time points of the study and *t*_sl_ is the shelf life.

## Results and discussion

### Characteristics of the materials produced

#### Freeze-dried material

The average water content in the final bottles of the FD material was 6.1 ± 0.7 % (*m*/*m*) as determined using volumetric Karl Fischer titration system (Metrohm, Herisau, CH). For a dry mass determination of the materials, the water content must be checked on a separate portion of the sample. The top particle size (*X*_90_) of the resulting powder was <400 μm as determined by laser diffraction (Symptec Helos Clausthal-Zellerfeld, DE). The fat content in the dry material is close to 15 % (as declared by the manufacturer considering the dry mass in the material). The mass loss in the freeze-dryer corresponds to about 81 % (*m*/*m*) which basically corresponds to the water content in the commercially available baby food.

#### Frozen material

The FR material had the characteristics of the commercially supplied material with respect to water content and particle size. The supplied material is essentially identical to routine samples with the exception of 9.5 h of very gentle mixing in a paste mixer with the addition of the spike in 29 mL acetonitrile. The fat content in the FR is 3.4 % as declared by the manufacturer. For both the FR and AC materials, it is essential that the water content is constant over a production series since any analyte should be certified as is.

#### Autoclaved material

The AC material is the same as the frozen material except for the final thermal treatment. Therefore, water content and particle size distribution are the same in both materials. The supplied material is identical to routine samples with the exception of 9.5 h of very gentle mixing in a paste mixer with the addition of the spike in 29 mL acetonitrile. Tests have shown that no mass loss took place in the autoclavation step. The fat content in the AC material is 3.4 % as declared by the manufacturer.

### Performance of the analytical procedure

**Fig. 2:**
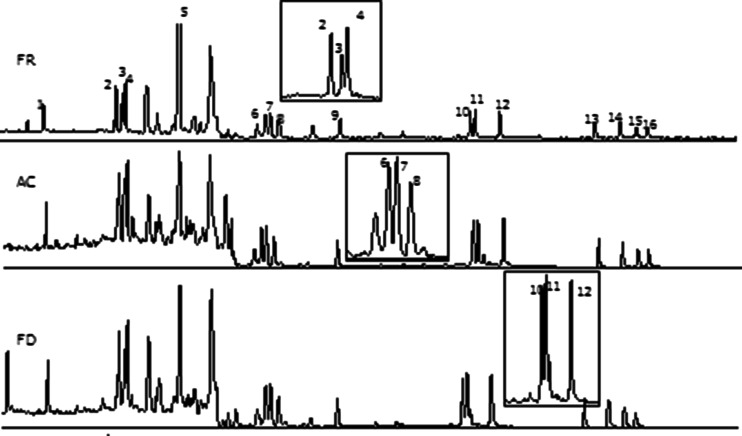
GC-MS chromatogram corresponding to frozen, autoclaved and freeze-dried materials. Identification of peaks: *1*, BcF; *2*, BaA; *3*, Chr; *4*, CPP; *5*, 5-MC; *6*, BbF; *7*, BkF; *8*, BjF; *9*, BaP; *10*, IcdP; *11*, DahA; *12*, BghiP; *13*, DalP; *14*, DaeP; *15*, DaiP; *16*, DahP

It was concluded during the development of the method that optimal results are obtained by the use of a DB-17HT column. This column provided sufficient separation of the target analytes. Closely eluting peaks such as CPP/Chr, BbF/BkF and IcdP/DahA were separated by a valley equal or minor to 20 % of the height of the smallest peak within the pair. The analytes were identified and confirmed by the following criteria: relative retention time (*t*_R_ in the sample/*t*_R_ in calibration solution) ≥0.99, presence of the quantification ion as well as one confirmation ion (Table 2) and relative qualifying ion abundance in sample within ±15 % of the analogous compound in the calibration solution. The criteria were met for all analytes down to a level comparable with the estimated LODs, except for 5-MC, for which an interference was observed, possibly because of the presence and co-elution of another MC isomer. Thus, the validation criterion regarding selectivity was not entirely fulfilled for this compound, and therefore it was not considered for subsequent studies. Figure 2 shows typical chromatograms from processed FR, AC and FD samples.

Calibration curves were obtained as described in “Method validation”. The statistical parameters calculated from least-square regression are presented in the Electronic supplementary material (ESM) in Table S1. The correlation coefficients (*r*) were >0.99, and the *p* values for the lack-of-fit test (*α* = 0.05) were larger than 0.05. In addition, the average RSD of RRFs obtained each day for each calibration level was lower than 5 % for all the analytes. Thus, the linearity of the analyte responses is confirmed over the tested range. LOD and LOQ values were extrapolated from the calibration curves to calculate absolute mass fraction values in a sample: 10 g wet sample for AC and FR materials and 2 g dry sample for the FD material. Blank samples of each material were spiked at these levels and analysed in order to verify that PAHs could easily be detected, identified and quantified at these mass fraction levels. As shown in ESM Table S1, LOQs for all considered PAHs were in the range 0.1–0.9 μg/kg for the AC and FR materials. In the case of the FD material, higher values, in the range 0.6–4.5 μg/kg, where obtained because of the water loss and pre-mass fraction after the freeze-drying process. The repeatability of the method ranged from 1.6 to 11.7 % in the three materials. Table 3 summarises the results obtained from recovery studies carried out at two mass fraction levels of each individual PAH in the three different materials. Satisfactory results in terms of recovery and RSD were obtained in most cases, although some differences were found depending on the material, especially for dibenzopyrenes. These high molecular mass PAHs (*m*/*z* 302) normally yield low analytical signal intensities [17]. For FR material, recoveries in the range 86–101 % with RSDs equal or lower than 9.0 % were obtained. Exceptions were CPR and CHR with recoveries of 112 % at 1 μg/kg and 113 and 108 % at 1.5 μg/kg, respectively. However, the excellent RSD obtained for his compounds in FR material (≤5.6 %) suggest that the method is suitable for the current CRM production feasibility study. In the case of the AC material, recoveries were in the range 83–101 % for all analytes, except for CPP and Chr for which a higher value (112 and 109 % respectively at 1 μg/kg; 113 and 117 %, respectively at 1.5 μg/kg) were obtained. Similarly than for the FR material, the RSD for these two compounds were considered good enough for the present study (≤6.1 %). RSDs for the rest of the compounds in the AC material were better than 9.8 %, except for DahP (12.7 and 16.7 % at 1 and 1.5 μg/kg concentration levels, respectively). As an exception, poor results were obtained for BghiP at 1 μg/kg mass fraction level (recovery of 113 % with an RSD of 51 %); however, they were assumed to be caused by experimental systematic errors as good results were obtained for the same compound in the same material at 1.5 μg/kg (recovery of 93 %, RSD 1.6 %). The FD material showed recoveries in the range 90–109 % for all the PAHs with some exceptions. For DalP and DaeP at 5 μg/kg mass fraction level, a yield higher than 120 % was found, while for DahP, it was 83 % at 5 μg/kg and 65 % at 7.5 μg/kg mass fraction levels (these results could be explained by the relatively higher RSD obtained for this compound). As mentioned before, the low analytical signal yielded by these late eluting compounds with high molecular mass, have a major influence on these results as they are peaks difficult to integrate. Recoveries of 117 and 110 % were obtained for Chr at low and high concentration level, respectively. However, good RSD (≤9.5 %) was obtained for this compound at both concentration levels, and therefore the method is assumed to provide reliable information for the purpose of the present study. Regarding the RSDs obtained for the rest of PAHs in the FD material, they were in the range 1.6–12.8 %, except for dibenzopyrene isomers (late eluting heavy compounds) with RSD varying from 14.2 to 22.8 %. To complete the studies, the stability of extracts was tested over a period of 4 weeks, showing no signs of degradation. Between the injections, the extracts were kept at +4 °C in the dark.

**Table 3:** Mean recoveries (%) and RSD (%, *n* = 6) obtained for the 15 + 1 EU priority PAHs at two different mass fraction levels in the three treated materials

PAH	Frozen material				Autoclaved material				Freeze-dried material			
	1 μg/kg		1.5 μg/kg		1 μg/kg		1.5 μg/kg		5 μg/kg		7.5 μg/kg	
	Recovery (%)	RSD (%)	Recovery (%)	RSD (%)	Recovery (%)	RSD (%)	Recovery (%)	RSD (%)	Recovery (%)	RSD (%)	Recovery (%)	RSD (%)
BcF	89	2.9	89	3.0	91	2.5	91	2.2	91	4.1	91	2.2
BaA	95	3.7	98	2.5	98	1.9	101	1.3	93	7.4	98	4.9
CPP	112	3.8	113	2.5	112	2.1	113	1.5	104	7.6	109	3.9
Chr	112	5.6	108	3.7	109	3.8	117	6.1	117	9.4	110	4.2
5-MC	–	–	–	–	–	–	–	–	–	–	–	–
BbF	100	5.3	100	4.9	99	8.4	100	2.3	92	2.1	91	2.3
BkF	90	5.2	95	1.7	90	6.8	91	4.4	96	1.9	97	1.6
BjF	100	2.8	101	3.8	100	4.9	99	2.7	94	2.4	95	3.3
BaP	97	3.4	96	3.8	91	6.4	97	1.7	95	6.6	95	5.0
IcdP	96	3.8	95	1.1	93	2.2	94	1.4	91	9.7	95	2.2
DahA	99	3.5	99	2.2	105	1.8	99	3.3	90	12.3	105	3.2
BghiP	94	3.8	92	3.2	113	51.0	93	1.6	91	4.1	94	3.2
DalP	92	4.1	93	6.3	94	8.9	89	9.4	131	4.7	100	14.2
DaeP	89	5.1	89	7.3	93	8.6	86	6.6	121	6.2	101	16.8
DaiP	90	5.8	89	7.7	91	7.6	81	9.6	102	9.2	93	16.4
DahP	88	6.6	86	9.0	95	12.7	83	16.7	83	20.0	65	22.8

### Homogeneity

The evaluation of the data for the homogeneity study (not shown) revealed that for all considered analytes, both the individual results and bottle means showed unimodal and, in most cases, normal distribution. A small number of bottle mean outliers (maximum one per compound) were identified for the different processed materials. As no technical reason could be identified for the occurrence of these outliers, all data were retained for statistical analysis in order to provide a conservative estimate of inhomogeneity for the materials.

A regression analysis was performed to detect possible trends regarding the filling or the analytical sequence. With the exception of BaA in the frozen matrix, no statistically significant trend was detected for PAHs in the filling sequence of neither FD, FR nor AC material. The degree of possible heterogeneity in each of the materials was evaluated through the estimation of uncertainties as described earlier. The outcome, summarised in ESM Table S2, does not show any major influence of the processing strategy concerning the homogeneity of the material, as most of the *u*_bb_ values are around or below 5 %. Regarding the variability within-bottle (*s*_wb_), values close to or even above 10 % were obtained in some cases. To reduce further this variability, an improved homogenisation step during processing or an increase of the sample intake during analysis could be envisaged prior to processing of a candidate reference material. Despite this effect, the overall results show the suitability of the three processing strategies tested for the preparation of sufficiently homogeneous candidate materials for PAHs in baby food.

### Stability

Analytical data obtained for each temperature and time scheme of the isochronous stability studies (results not shown) were evaluated as described earlier. Some outliers were found but retained since no technical reason for their exclusion could be justified. The slope of the regression line of the PAH mass fraction *vs*. time was evaluated for statistical significance in order to detect any trend of instability in the material. Table S3 in the ESM summarises the evaluation of the short-term stability study for the three materials. None of the analyte/matrix combinations investigated showed a statistically significant trend at low or medium temperatures (4 and 18 °C for the AC and FR materials and −20 and 4 °C for the FR material). For the AC material at 4 °C, BjF data showed a significant slope (*α* = 0.01), which was possibly an analytical artefact as no significance was observed at 18 °C or even at 60 °C. A significant slope was obtained for BkF at 60 °C in the AT material and Chr in the FD material. From the short-term stability study, it can be concluded that AC and FD materials should be shipped with cooling elements and the FR material in dry ice.

**Fig. 3:**
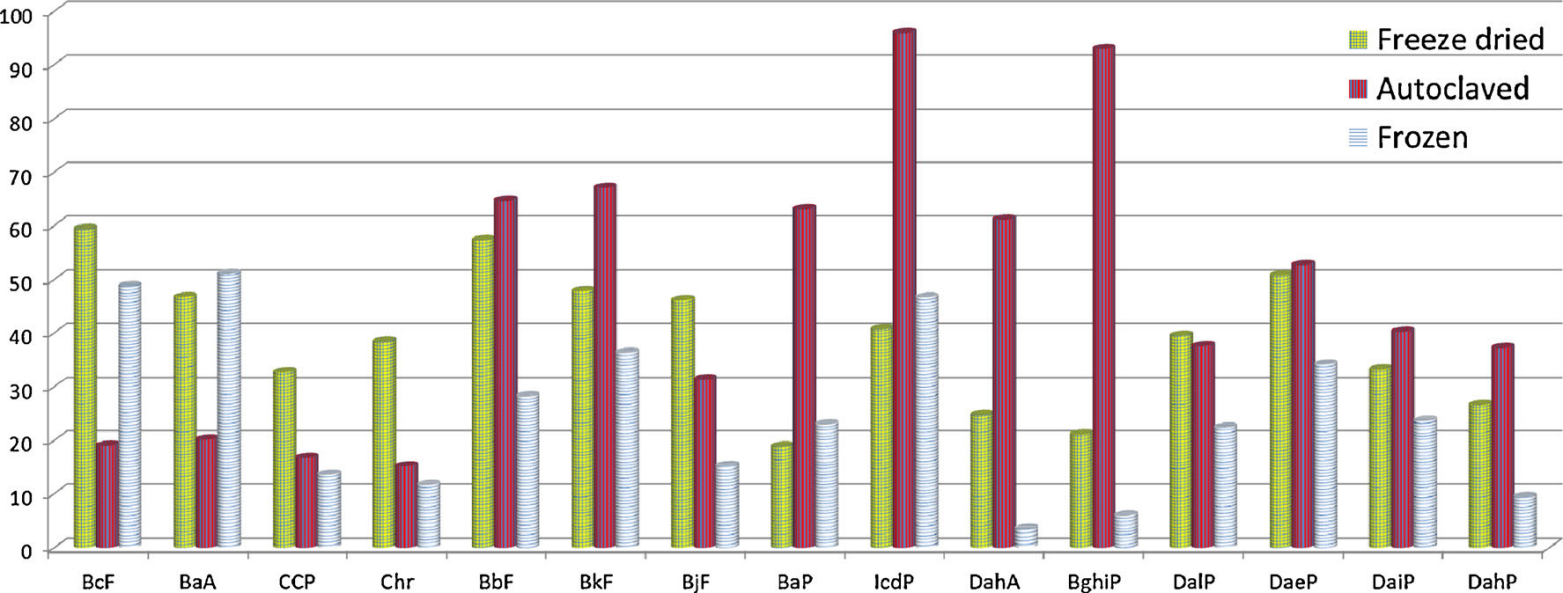
Shelf lives (months) predicted for a target uncertainty of 5 % for each considered PAH in the frozen, autoclaved and freeze-dried materials

The slopes and statistical parameters associated with the long-term stability study are presented in Table 4. In this case, the AC material tested at 4 and 18 °C for 18 months, showed degradation only for DahP at both temperatures (*α* = 0.01). A significant slope was detected for DahA at 18 °C, indicating losses for this analyte when stored under these conditions. For the FD material, five PAHs (CPP, BaP, DalP, DaeP, DahP) showed a statistically significant slope at 18 °C. Furthermore, DahP exhibited instability as well at 4 °C. In the case of BbF, although showing a significant slope at 4 °C, results suggested stability of this compound when the FD material is stored at 18 °C. None of the analytes showed significant trend in the frozen material at −20 °C (*α* = 0.01). In order to provide a more general picture about the stability of the analytes between the different materials, data were re-evaluated at 95 % of confidence levels. No significant changes were found compared with results at 99 % confidence interval except in very few cases. BjF and DaiP exhibited significant trend in the AC material at 4 °C (*α* = 0.05). However, results for BjF proved stability for this compound when the AC material is stored at 18 °C. On the other hand, DaiP showed instability at 18 °C in the AC material, similarly to the results obtained when data were scrutinised at 99 % confidence level. In the case of the FD material, the only difference found when data were evaluated at 95 % confidence level concerned only DaiP, which showed significant trends at both temperatures (4 and 18 °C), therefore these results show losses of this compound in the FD material. No differences were found for FR at −20 °C when results for the LTS study were scrutinised at 95 % confidence interval and were compared with those scrutinised at 99 % confidence interval. For the sake of comparison, the approximate shelf lives for each considered PAH in the FR, AC and FD materials were predicted for a target uncertainty of 5 %. As can be seen in Fig. 3, for the majority of the analytes, adequate stability was obtained for the freeze-dried material stored at 4 °C. For a CRM producer, higher temperatures (ideally above zero) facilitate the storage and distribution of the material. However, freeze-dried materials are provided in a physical form that requires reconstitution and which could potentially change the properties of the matrix after reconstitution.

**Table 4:** Linear regression and statistical parameters associated to long-term stability

PAH	Outlier (*α* = 0.01)	Slope (%/months)	Significant slope (*α* = 0.01)	*u*_lts_ (%)	Outlier (*α* = 0.01)	Slope (%/months)	Significant slope (*α* = 0.01)	*u*_lts_ (%)	Outlier (*α* = 0.01)	Slope (%/months)	Significant slope (*α* = 0.01)	*u*_lts_ (%)	Outlier (*α* = 0.01)	Slope (%/months)	Significant slope (*α* = 0.01)	*u*_lts_ (%)	Outlier (*α* = 0.01)	Slope (%/months)	Significant slope (*α* = 0.01)	*u*_lts_ (%)
	Autoclaved at 4 °C				Autoclaved at 18 °C				Frozen at −20 °C				Freeze dried at 4 °C				Freeze dried at 18 °C			
BcF	No	−0.17	No	4.74	No	0.16	No	3.88	No	0.00	No	1.85	No	−0.08	No	1.52	No	−0.17	No	2.22
BaA	No	−0.28	No	4.46	No	0.09	No	4.29	No	−0.10	No	1.76	No	−0.12	No	1.93	No	−0.05	No	2.41
CPP	No	−0.25	No	5.37	No	−0.08	No	5.15	1	−0.27	No	6.63	No	−0.28	No	2.75	No	−0.44	Yes	2.59
Chr	1	−0.32	No	5.94	No	0.00	No	3.77	1	0.31	No	7.73	No	−0.09	No	2.35	1	0.09	No	3.18
5-MC	–	–	–	–	–	–	–	–	–	–	–	–	–	–	–	–	–	–	–	–
BbF	No	0.07	No	1.39	1	0.14	No	1.31	No	−0.15	No	3.19	No	−0.27	Yes	1.57	No	−0.09	No	4.58
BkF	No	−0.07	No	1.34	1	0.07	No	1.83	No	−0.14	No	2.48	No	−0.02	No	1.88	No	0.00	No	1.92
BjF	1	0.40	No	2.87	1	0.16	No	2.50	No	0.00	No	5.92	1	−0.09	No	1.95	No	−0.09	No	1.42
BaP	No	−0.10	No	1.43	No	0.00	No	1.59	1	−0.10	No	3.92	1	−0.21	No	4.77	No	−0.44	Yes	2.15
IcdP	No	0.00	No	0.94	No	0.08	No	1.27	1	1.70	No	1.93	1	−0.05	No	2.21	No	−0.17	No	1.85
DahA	1	1.10	No	1.47	No	−0.64	Yes	2.07	1	0.61	No	25.57	No	0.32	No	3.64	1	−0.25	No	4.21
BghiP	No	−0.08	No	0.97	1	−0.08	No	5.17	1	0.40	No	15.15	1	−0.33	No	4.26	No	−0.46	No	5.86
DalP	No	0.18	No	2.40	No	0.28	No	2.90	1	0.30	No	4.02	No	−0.02	No	2.28	No	−0.60	Yes	3.27
DaeP	No	0.10	No	1.71	No	0.19	No	1.97	2	0.00	No	2.64	No	−0.02	No	1.77	No	−0.36	Yes	1.68
DaiP	No	−0.35	No	2.24	No	−0.35	Yes	1.54	No	−0.34	No	3.80	1	−0.43	No	2.71	1	−0.96	No	6.55
DahP	1	0.40	Yes	2.42	No	−0.52	Yes	1.99	1	0.20	No	9.59	No	−1.61	Yes	3.38	No	−2.50	Yes	4.58

*u*_*lts*_ uncertainty of long-term stability

One purpose of this study was to be able to prepare a sufficiently stable material that can be stored at a temperature above 0 °C. It would also be advantageous if the material could be supplied in a physical form as close as possible to routine samples. Results shown in Fig. 3 for the AC material stored at 4 °C indicate a notable improvement on the stability for a number of compounds, compared with the other two processing techniques. Particularly relevant is the good stability of BaP and BbF, both compounds are included in the EU legislation for PAHs in foodstuff [12]. On the other hand, the stability of the lighter compounds, e.g. BcF, BaA, CPP and Chr, is reduced, or in the best case similar in comparison with the other two processing techniques.

## Conclusions

During the feasibility study for the production of a CRM for 15 + 1 EU PAHs in baby food, the potential of autoclaving has been investigated and compared with two processing techniques classically employed for the production of food CRMs, namely freezing and freeze drying. Analyses performed with a GC-MS method developed and validated in-house, indicated an acceptable level of heterogeneity for the PAHs investigated in each of the three materials. 5-MC was excluded from the studies as the validation criterion regarding the selectivity was not completely fulfilled for this compound. At storage temperatures of 4 °C (for autoclaved and freeze-dried) and at −20 °C (for the frozen material), PAH degradation was not statistically significant for a period of 18 months, with a few exceptions. However, the extrapolation of the results for the estimation of the shelf life for the baby food preparations indicate an increased stability of the PAHs in the autoclaved material compared with the freeze-dried material, with the exception of the lighter PAHs (BcF, BaA, CPP and Chr). The stability of these compounds is negatively influenced by the autoclaving process. Both autoclaved and freeze-dried materials exhibit increased stability in comparison with the frozen material with exception of the two lightest compounds. In summary, freeze drying and autoclaving were found to be suitable processing techniques for the production of a candidate CRM for PAHs in baby food. For the lightest PAHs, autoclaving conditions would require further investigation for improved results. Finally, it is important to realise that an autoclaved wet paste resembles routine samples to a much higher degree than a freeze-dried powder. Therefore, processing methodologies involving thermal sterilisation are very promising for the development of a matrix reference material for PAHs in a processed food matrix similar to routine samples.

## Electronic supplementary material

Below is the link to the electronic supplementary material.

ESM 1 (PDF 25 kb)
