# Supplementary material for: PAHs in baby food: assessment of three different processing techniques for the preparation of reference materials
Source: Anal Bioanal Chem. 2015 Feb 3;407(11):3069–81. doi: 10.1007/s00216-015-8490-z (PMC4383830; doi:10.1007/s00216-015-8490-z)
Supplement: Supplementary file 1 — (PDF 25 kb) [file 216_2015_8490_MOESM1_ESM.pdf]

Analytical and Bioanalytical Chemistry

Electronic Supplementary Material

PAHs in baby food: Assessment of three different processing techniques for the preparation of reference materials

José Fernando Huertas-Pérez, Luisa R. Bordajandi, Berit Sejerøe-Olsen, Håkan Emteborg, Andrea Baù, Heinz Schimmel, Marta Dabrio

Table S1 LODs, LOQs and external calibration equation

PAH	LOD [µg/kg]	LOQ [µg/kg]	LOD [µg/kg]	LOQ [µg/kg]	RSD (%) [b]			Calibration Equation [a]	$S_{R,c}$	r	Lack of Fit P-value (α=0.05)
	AC and FR materials		*FD material*		*FR material*	*AC material*	*FD material*				
BcF	0.1	0.3	0.7	1.3	4.0	9.3	2.9	Y = 0.047 + 1.376 X	0.040	0.998	0.730
BaA	0.3	0.4	1.3	2.1	4.0	8.6	3.6	Y = -0.025 + 1.281 X	0.035	0.998	0.729
CPP	0.6	0.9	2.9	4.5	6.7	10.3	5.7	Y = -0.043 + 1.250 X	0.030	0.999	0.603
CHR	0.3	0.5	1.6	2.7	7.3	8.6	5.4	Y = -0.016 + 1.128 X	0.041	0.996	0.989
5-MC	-	-	-	-	-	-	-	-	-	-	-
BbF	0.1	0.2	0.6	1.0	6.7	2.5	3.6	Y = -0.033 + 1.029 X	0.026	0.998	0.147
BkF	0.1	0.2	0.7	1.2	5.8	1.9	3.5	Y = -0.022 + 0.958 X	0.024	0.998	0.206
BjF	0.1	0.2	0.7	1.2	11.7	1.8	2.8	Y = -0.031 + 0.984 X	0.047	0.995	0.974
BaP	0.1	0.2	0.6	1.0	8.7	2.5	3.3	Y = -0.011 + 0.958 X	0.030	0.997	0.314
IcdP	0.1	0.1	0.4	0.6	2.9	1.8	3.0	Y = 0.008 + 0.995 X	0.018	0.999	0.823
DahA	0.1	0.2	0.5	0.9	8.5	3.0	4.3	Y = 0.013 + 1.174 X	0.031	0.998	0.774
BghiP	0.1	0.2	0.4	0.8	8.7	1.6	5.4	Y = 0.006 + 0.997 X	0.031	0.997	0.392
DalP	0.5	0.8	2.6	4.1	3.8	4.8	4.5	Y = 0.052 + 2.013 X	0.201	0.992	0.355
DaeP	0.4	0.7	2.2	3.5	3.5	3.1	3.2	Y = -0.107 + 1.996 X	0.134	0.991	0.795
DaiP	0.4	0.6	1.8	2.8	6.6	3.6	7.0	Y = 0.011 + 1.119 X	0.066	0.992	0.219
DahP	0.4	0.6	1.8	2.8	11.4	5.5	7.0	Y = -0.070 + 1.160 X	0.072	0.991	0.182

[a)] Obtained from triplicate injection of the standard solutions. Y: area ratio analyte/I.S.; X: concentration ratio analyte/I.S. Results are shown for 1 day and are representative for all validation days (n=3)

[b)] 6 samples processed and injected in duplicate (n=12). $S_{R,c}$: Regression standard deviation

r: Correlation coefficient

Table S2 Estimated uncertainty contributions from homogeneity study

PAH	Autoclaved batch			Frozen batch			Freeze dried batch		
	s_{wb} [a] (%)	s_{bb} [b] (%)	u^*_{bb} [c] (%)	s_{wb} [a] (%)	s_{bb} [b] (%)	u^*_{bb} [c] (%)	s_{wb} [a] (%)	s_{bb} [b] (%)	u^*_{bb} [c] (%)
BcF	10.0	n.c.	3.4	6.1	2.7	2.2	4.5	1.9	2.3
BaA	4.8	12.9	1.7	3.6	3.9	1.3	6.2	n.c.	3.1
Chr	4.7	n.c.	1.6	4.5	2.2	1.6	3.8	0.3	1.9
CCP	4.9	n.c.	1.7	5.5	2.9	1.9	5.3	n.c.	2.6
5MC	-	-	-	-	-	-	-	-	-
BbF	5.1	3.7	1.8	4.9	n.c.	1.8	4.3	n.c.	2.1
BkF	5.0	3.4	1.7	3.5	n.c.	1.2	4.3	2.4	2.2
BjF	5.1	1.8	2.0	4.0	1.1	1.4	4.9	3.3	2.5
BaP	3.5	5.5	1.2	5.7	n.c.	2.0	3.3	2.3	1.7
IcdP	10.8	n.c.	3.7	2.4	3.3	0.8	4.8	1.1	2.4
DahA	14.7	13.0	5.1	8.6	2.3	3.1	4.7	4.3	2.4
BghiP	11.4	n.c.	3.9	3.4	4.9	1.2	4.4	2.0	2.2
DalP	9.3	n.c.	3.2	8.1	7.6	2.9	10.6	n.c.	5.3
DaeP	9.8	n.c.	3.4	8.2	5.7	2.9	10.2	n.c	5.1
DaiP	9.6	n.c.	3.3	7.3	5.7	2.6	9.6	n.c.	4.8
DahP	8.8	n.c.	3.0	8.3	7.5	2.9	10.4	n.c.	5.2

[a] s_{wb}, standard deviation of within bottle variation

[b] s_{bb}, standard deviation of between-bottle variation

[c] u^*_{bb}, defined as uncertainty due to possible heterogeneity that can be hidden by the method repeatability

[d] u_{outl}, alternative uncertainty estimation in case of outlying bottle means or a trend in the filling sequence

n.c., cannot be calculated (because of $MS_{between} < MS_{within}$ in ANOVA)

Table S3 Linear regression and statistical parameters associated to short-term stability

PAH	Outlier (α=0.01)	Slope [%/week]	Significant slope (α=0.01)	u_{sts} [a] [%]	Outlier (α=0.01)	Slope [%/week]	Significant slope (α=0.01)	u_{sts} [a] [%]	Outlier (α=0.01)	Slope [%/week]	Significant slope (α=0.01)	u_{sts} [a] [%]
		Autoclaved at 4ºC				*Autoclaved at 18ºC*				*Autoclaved at 60ºC*		
BcF	No	-1.64	No	5.01	No	0.77	No	4.37	No	1.97	No	4.34
BaA	2	-2.26	No	7.28	No	0.52	No	2.48	No	1.87	No	2.80
Chr	No	0.61	No	2.52	No	0.60	No	2.35	No	-0.28	No	2.43
CCP	No	-1.22	No	2.31	No	0.42	No	2.22	No	1.80	No	2.76
5MC	-	-	-	-	-	-	-	-	-	-	-	-
BbF	No	-1.35	No	3.45	1	1.54	No	3.21	No	2.08	No	6.78
BkF	No	-1.68	No	3.04	No	0.28	No	2.28	No	2.03	**Yes**	2.36
BjF	No	12.82	**Yes**	8.84	1	0.20	No	6.53	No	3.23	No	3.38
BaP	No	-1.91	No	3.18	1	4.24	No	7.99	1	6.15	No	59.70
IcdP	No	-1.58	No	5.29	No	0.06	No	5.98	No	0.79	No	6.62
DahA	1	-0.16	No	10.63	No	-0.09	No	5.34	1	2.75	No	7.47
BghiP	No	-0.88	No	5.55	No	-0.53	No	6.23	No	1.34	No	7.31
DalP	No	-0.59	No	4.44	No	1.37	No	4.57	No	3.43	No	4.42
DaeP	No	-1.18	No	4.73	No	1.27	No	4.29	No	1.62	No	4.11
DaiP	No	-1.16	No	4.64	No	0.62	No	3.98	No	2.43	No	3.80
DahP	No	-1.11	No	4.31	No	0.88	No	3.88	No	2.85	No	3.78
		Frozen at -20ºC				*Frozen at 4ºC*				*Frozen at 18ºC*		
BcF	No	-0.21	No	3.67	No	2.43	No	5.57	No	1.12	No	3.47
BaA	No	0.07	No	2.88	No	1.42	No	4.45	No	0.42	No	2.58
Chr	No	-0.76	No	3.39	No	1.63	No	4.77	No	0.20	No	2.88
CCP	No	0.30	No	2.73	No	1.27	No	4.55	No	-0.38	No	2.48
5MC	-	-	-	-	-	-	-	-	-	-	-	-
BbF	No	-0.38	No	2.58	No	1.40	No	4.06	No	-0.19	No	2.68
BkF	No	0.37	No	1.90	No	1.04	No	4.03	No	0.24	No	2.45
BjF	No	0.82	No	2.31	2	0.80	No	4.14	No	-0.12	No	2.22
BaP	No	-0.24	No	2.87	No	0.87	No	4.06	No	0.41	No	3.06
IcdP	No	-0.71	No	2.16	2	0.90	No	3.87	No	0.27	No	1.91
DahA	No	-0.85	No	4.90	No	1.50	No	5.18	No	1.40	No	5.02
BghiP	No	-0.04	No	3.19	No	1.75	No	4.39	No	0.26	No	2.13
DalP	No	-0.78	No	6.00	No	1.52	No	5.83	No	2.46	No	4.41
DaeP	No	-1.23	No	5.45	No	0.92	No	5.92	No	2.41	No	4.97
DaiP	No	-1.29	No	5.01	No	0.75	No	5.81	No	1.65	No	4.74
DahP	No	-1.34	No	6.04	No	1.23	No	5.66	No	1.82	No	4.57
		Freeze dried at 4ºC				*Freeze dried at 18ºC*				*Freeze dried at 60ºC*		
BcF	No	0.12	No	3.30	No	-0.43	No	4.47	No	-1.58	No	4.98
BaA	No	0.00	No	3.78	No	-0.18	No	3.98	1	-1.38	No	4.93
Chr	No	0.01	No	3.10	No	0.10	No	3.95	No	-7.07	**Yes**	6.06

CCP	No	0.22	No	2.60	No	0.01	No	3.42	No	-0.74	No	4.94
5MC	-	-	-	-	-	-	-	-	-	-	-	-
BbF	No	-0.68	No	2.64	No	0.62	No	2.87	1	-0.43	No	4.38
BkF	No	0.40	No	3.34	No	-0.36	No	2.46	1	-0.63	No	5.10
BjF	No	0.28	No	3.97	No	-0.94	No	2.91	1	-0.61	No	5.86
BaP	No	0.05	No	2.70	No	-0.74	No	3.02	1	0.18	No	4.48
IcdP	No	-0.66	No	3.28	No	-0.78	No	3.39	1	-1.18	No	5.62
DahA	No	-0.52	No	4.27	No	0.67	No	4.51	1	-1.07	No	5.77
BghiP	No	-0.47	No	3.24	No	-1.78	No	3.91	1	-2.62	No	5.85
DalP	No	-1.18	No	6.25	No	-0.43	No	4.06	No	-1.92	No	7.74
DaeP	No	-0.34	No	6.13	No	-0.08	No	3.64	2	-2.48	No	7.69
DaiP	No	-2.62	No	6.41	No	-0.40	No	4.74	No	-3.59	No	8.21
DahP	No	-2.14	No	6.64	No	-0.40	No	3.90	No	-2.46	No	8.09

[a] u_{sts}, uncertainty of short-term stability
